# Burden and Determinants of Smoking among Prisoners with Respiratory Tract Infection: A Cross-Sectional Study of Nine Major Prison Setups in Northern Ethiopia

**DOI:** 10.1371/journal.pone.0168941

**Published:** 2016-12-28

**Authors:** Semaw Ferede Abera, Kelemework Adane

**Affiliations:** 1 School of Public Health, College of Health Sciences, Mekelle University, Mekelle, Ethiopia; 2 Kilte Awlaelo Health and Demographic Surveillance Site, Mekelle, Ethiopia; 3 Department of Medical Microbiology and Immunology, College of Health Sciences, Mekelle University, Mekelle, Ethiopia; Liverpool School of Tropical Medicine, UNITED KINGDOM

## Abstract

**Background:**

Morbidity, mortality and a wide range of associated risk factors are disproportionately clustered among prisoners compared to the general population. Smoking is one of the risk factors for the increased burden of unfavorable health outcomes particularly among prisoners. However, little is known about the level and determinants of smoking among the incarcerated population in Ethiopia.

**Methods:**

We collected data from 738 prisoners in nine major prison setups in Tigray region by nurses and clinical officers. Data were entered in to Epi Data 3.1 and exported to stata 13.0 for cleaning and further analysis. Multivariable logistic regression model was fitted to identify determinants of smoking at p value of less than 5%.

**Result:**

The prevalence of smoking was 21 per cent (95%CI = 18.2%, 24.1). Urban residence (AOR = 2.15; 95%CI = 1.20, 3.84), previous history of incarceration (AOR = 1.91; 95%CI = 1.08, 3.40) and alcohol use before incarcerated (AOR = 4.20; 95%CI = 2.57, 6.87) were significantly associated with risk of smoking. In contrast, risk of smoking was significantly lower for farmers (AOR = 0.20; 95% CI = 0.08, 0.49), prisoners with family support (AOR = 0.52; 95% CI = 0.32, 0.87) and for those who were jailed in Shire prison site (AOR = 0.43; 95%CI = 0.20, 0.95).

**Conclusion:**

Our work clearly indicates that the observed smoking prevalence calls for the need of comprehensive and interdisciplinary interventions targeting prisoners.

## Background

Prisoners are mostly exposed to disproportionately high levels of different risk factors, morbidity and mortality compared to the general population [1–6]. Mortality after release from prison was also reported to be higher, particularly during the early period of release [1,7,8]. Smoking has been reported to be a dominant risk factor associated with increased risk of death among prisoners and a smoking ban in prisons was strongly associated with substantially reduced mortality [9–12].

A number of researchers have reported that the burden of smoking was high among prisoners, including among former prisoners [5,6,13–16]. On the other hand, multiple studies have revealed that lower educational attainment, stress, HIV comorbidity, previous history of incarceration, mass incarceration, history of difficult life circumstances, engagement in illegal activities, adverse childhood events and current personality traits were the significant determinants of smoking among prisoners [13–18].

However, most of the studies are from high income countries, and prison health studies are often marginalized by researchers from developing countries despite its critical public health importance to both the inmates and their community [19]. Similarly, data about the burden and determinants of smoking based on prisoners are scarce to inform decision makers in the health sector in developing countries, particularly in Ethiopia.

Therefore, in this paper, we aimed to determine the burden and determinants of smoking among prisoners incarcerated in nine major prisons in Tigray region, Ethiopia.

## Methods

### Study Setting and Period

We conducted a quantitative cross-sectional study from August 2013 to February 2014 in all the nine major prison setups, namely Mekelle, Wukro, Adigrat, Axum, Adwa, Shire, Humera, Maichew and Alamata. All the eight prison sites are located in the major towns of Tigray region and Mekelle prison is located in Mekelle city, the region’s capital city. During the study period, there were 9, 326 prisoners in these nine prison sites. According to the updated report data from the region’s Correction Office, there were 710 prisoners in Alamata, 786 in Maichew, 2, 557 in Mekelle, 602 in Wukro, 1, 093 in Adigrat, 537 in Adwa, 653 in Axum, 1, 513 in Shire and 875 in Humera.

### Sample Size Calculation and Sampling Method

The sample size was determined assuming a smoking prevalence of 50%, precision of 5%, a confidence interval of 95%, multiplying by design effect of 2 and adding a 5% non-response rate. It is valuable to assume that decimals are rounded up to ensure minimum required sample. Using a single proportion formula, accounting the abovementioned parameters, it translates to a sample size of 809.

A proportional stratified sampling technique was used to select strata specific study participants. First, we proportionally stratified the sample size by prison site, based on their total number of prisoners during the study period. The stratum specific coefficient, 809 divided by 9326, multiplied by the respective site specific prison population gave us the sample participants needed in each site. Then, we applied a convenient sampling method to select strata-specific actual participants, who fulfilled the inclusion criteria, of our study.

### Data Collection and Management

Data on smoking and potential covariates were collected as part of our tuberculosis (TB) study. The main inclusion criteria were a cough lasting two or more weeks and one TB suggestive symptom, such as night sweating, chest pain and/or weight loss.

In each prison setup, experienced clinical officers and nurses screened and collected data, respectively. The outcome of interest to us was current smoking status. A pre-defined questionnaire including questions on current smoking status, other lifestyle profiles before and after incarceration (such as khat and alcohol consumption), individual socio-demographic characteristics, family support, HIV sero-status, previous history incarceration and prison-related factors was used to collect data from the prisoners. Current smoking was defined as smoking in the past 12 months. In this article, sentence status of a given prisoner means a given prisoner is already found guilty and is in jail, but whether he/she has already known his/her final decree of punishment as ordered by the judge or not.

The collected data were entered into Epi Data version 3.1 and exported to Stata version 13.0 for cleaning and further analysis.

### Statistical Analysis

Descriptive data summary statistics, tables and figures were used to summarize and present our findings. Chi-square test was used to assess the association of each independent covariate with smoking. A bivariate logistic regression analysis was performed and covariates which showed a p-value of <25% were considered to the multivariable logistic regression model to identify the determinants of smoking, using a p-value of <5% to declare statistical significance. A multicollinearity problem was diagnosed using variance inflation factor (VIF) at VIF level of greater than ten.

### Ethical Considerations

The Ethical Review committee of the College of Health Sciences in Mekelle University approved the study and the written informed consent. Individual participant based signed was obtained from each participant. To inform illiterate participants, our data collectors gave individual based clarification by reading the approved written consent for. Moreover, participants were informed to leave the study at any time if they did not want to participate or to exempt a question if they did not want to answer. We guaranteed them that there would be no harmful consequence of making such a decision. Once the consent procedure was done, signed informed consent was obtained from all participants. Moreover, all participants who had any health problem, who were not treated or who were treated but not improved, were linked to the prison health staff. Those prisoners who needed more care were referred to the nearby health centers and hospitals after discussing with the respective prison staff.

## Results

### Socio-Demographic Characteristics of Prisoners with Respiratory Infection

The overall response rate of our study was 91.2 per cent. Seventy-one (8.8%) participants did not disclose their smoking status. The non-respondent participants were a bit older, with a median age of 32 years, interquartile range (IQR) of 25–40 years, compared to those who responded. Explored according to other characteristics, 15.5 per cent were not sentenced, 71.8 per cent were from rural areas and 77.1 per cent were receiving family support. Moreover, 68.6 per cent were farmers, 14.1 per cent had a history of prior incarceration, and two were HIV positive. Surprisingly, 18.3 per cent and 66.2 per cent of the total non-respondents did not disclose their alcohol drinking habit and khat chewing status, respectively.

The median age of the prisoners was 29 years with an interquartile range of 24–39 years. Half of the prisoners were married, while more than a quarter of participants were illiterate. About half of the participants were not getting family support and almost all (721: 97.83%) of the participants were male (Table 1).

**Table 1:** Socio-demographic characteristics of prisoners with respiratory infection by smoking status in Tigray region in 2013/2014, northern Ethiopia (n = 738).

Socio-demographic characteristics	Smokers (%)	Non-smokers (%)	Total (%)	p-value*
**Sex**				0.311
** Female**	5 (31.25)	11(68.75)	16 (2.17)	
** Male**	150 (20.80)	571 (79.20)	721 (97.83)	
**Residence before jail**				<0.001
** Rural**	36 (9.14)	358 (90.86)	394 (53.9)	
** Urban**	117 (34.72)	220 (65.28)	337 (46.1)	
**Marital status**				<0.001
** Married**	53 (14.64)	309 (85.36)	362 (49.59)	
** Single**	86 (27.13)	231 (72.87)	317(43.42)	
** Divorced/widowed**	15 (29.41)	36 (70.59)	51(6.99)	
**Education**				<0.001
** Illiterate**	17 (8.85)	175 (91.15)	192 (26.02)	
** Primary**	74 (20.85)	281 (79.15)	355 (48.10)	
** Secondary**	50 (34.48)	95 (65.52)	145 (19.65)	
** College and above**	14 (30.43)	32 (69.57)	46 (6.23)	
**Occupation**				<0.001
** Civil servant**	22 (25.58)	64 (74.42)	86 (11.65)	
** Farmer**	22 (6.34)	325 (93.66)	347 (47.02)	
** Merchant**	15 (35.71)	27 (64.29)	42 (5.69)	
** Daily laborer**	44 (38.94)	69 (61.06)	113 (15.31)	
** Student**	27 (30.34)	62 (69.66)	89 (12.06)	
** Driver/soldier& Others**	25 (40.98)	36 (59.02)	61 (8.27)	
**Family support**				0.004
** No**	94 (25.47)	275 (74.53)	369 (50.27)	
** Yes**	61 (16.71)	304 (83.29)	365 (49.73)	

* Indicates p-value from analysis using chi-square test

### Lifestyle and Prison-Related Characteristics of Prisoners with Respiratory Infection

About 15 per cent of the participants had a history of previous incarceration and more than half of them had been arrested twice or more. Moreover, more than 87 per cent of the prisoners were already sentenced. About 14 per cent of the prisoners reported that they had been admitted to hospital since joining the prison and nearly four per cent of the total prisoners reported having a known comorbidity on top of their coughing symptom (Table 2).

**Table 2:** Shows the lifestyle and prison related characteristics of prisoners with respiratory infection by smoking status in Tigray region in 2013/2014, northern Ethiopia (n = 738).

Lifestyle and prison related characteristics	Smokers (%)	Non-smokers (%)	Total (%)	p-value*
**History of jail**				<0.001
** No**	116 (18.68)	505 (81.32)	621 (84.15)	
** Yes**	39 (33.33)	78 (66.67)	117 (15.85)	
**Frequency of previous jail**				0.137
** 1**	18 (32.73)	37(67.27)	55 (47.01)	
** > = 2**	21 (33.87)	41(66.13)	62(52.99)	
**Comorbidity**				0.478
** No**	140 (21.12)	523 (78.88)	663 (96.23)	
** Yes**	7 (26.92)	19 (73.08)	26 (3.77)	
**Ever hospitalized**				0.907
** No**	129 (21.57)	469 (78.43)	598 (86.29)	
** Yes**	21 (22.11)	74 (77.89)	95 (13.71)	
**HIV sero-status**				0.291
** No**	148 (20.82)	563 (79.18)	711 (98.34)	
** Yes**	4 (33.33)	8 (66.67)	12 (1.66)	
**Health education**				0.303
** No**	33 (24.81)	100(75.19)	133 (18.95)	
** Yes**	118(20.74)	451 (79.26)	569 (81.05)	
**Sentence status**				0.581
** Not sentenced**	22 (23.16)	73 (76.84)	95 (12.87)	
** Sentenced**	133 (20.68)	510 (79.32)	643 (87.13)	
**Multiple sexual partner**				<0.001
** No**	69(15.13)	387 (84.87)	456 (64.68)	
** Yes**	84 (33.73)	165 (66.27)	249 (35.32)	
**Alcohol use before jailed**				<0.001
** No**	49 (12.28)	350 (87.72)	399 (58.42)	
** Yes**	99 (34.86)	185 (65.14)	284 (41.58)	
**Duration of jail in years**				0.471
** <1**	52 (19.33)	217 (80.67)	269 (36.45)	
** 1–2**	29 (23.20)	96 (76.80)	125 (16.94)	
** 2–3**	44 (24.18)	138 (75.82)	182 (24.66)	
** > = 3**	30 (18.52)	132 (81.48)	162 (21.95)	

* Indicates p-value from analysis using chi-square test.

Additionally, more than 35 per cent of the participants reported a history of multiple sexual partners before being jailed. More than four-fifths of the participants reported that health education was given by nurses at their prison site and nearly half (46.61%) of the total participants had been imprisoned for two years and above (Table 2). In this study, the prevalence of culture confirmed TB cases was 3.9 per cent (95% CI = 2.7%, 5.6%).

**Fig 1:**
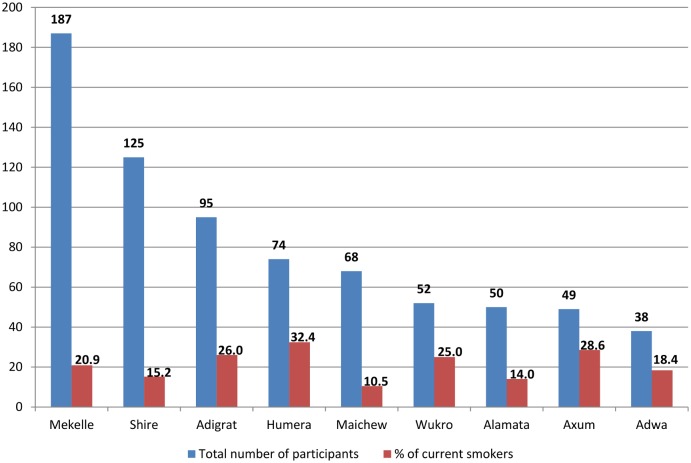
Shows the total number of participant prisoners with the percentage of smoking by prison site in Tigray region in 2013/2014, Ethiopia.

The number of prisoners, who had respiratory tract infection and who participated in our study, ranged from a minimum of 38 in Adwa to a maximum of 187 in Mekelle. The prevalence of smoking among prisoners with respiratory infection was 21 per cent (95% CI = 18.2%-24.1%). However, the prevalence of smoking showed variation from site to site, ranging from a minimum prevalence of 10.5 per cent in Maichew to a maximum of 32.4 per cent in Humera (Fig 1).

### Determinants of Smoking among Prisoners with Respiratory Infection

In this study, we found that Shire prison site (AOR = 0.43; 95%CI = 0.20, 0.95), family support (AOR = 0.52; 95% CI = 0.32, 0.87) and farmer occupation (AOR = 0.20; 95%CI = 0.08, 0.49) were significant determinants associated with lower odds of smoking among the prisoners (Table 3).

**Table 3:** Determinants of smoking among adult prisoners with respiratory infection in Tigray region in 2013/2014, Ethiopia (n = 738).

Variables		COR(95% CI)	p-value	AOR(95% CI)	P-value
Prison Site	Mekelle	1.00		1.00	
	Adwa	0.86(0.35, 2.09)	0.735	1.09 (0.35, 3.42)	0.887
	Alamata	0.62 (0.26, 1.48)	0.280	1. 43 (0.48, 4.23)	0.523
	Maichew	0.44 (0.19, 1.05)	0.063	0.48 (0.16, 1.49)	0.206
	Adigrat	1.34(0.75, 2.38)	0.324	1.04 (0.49, 2.22)	0.910
	Humera	1.82(1.00, 3.32)	0.051	1. 82 (0.79, 4.18)	0.158
	Shire	0.68(0.37, 1.24)	0.210	0.43 (0.20, 0.94)	0.034*
	Wukro	1.27 (0.62, 2.60)	0.522	0.68 (0.26, 1.75)	0.422
	Axum	1.52(0.74, 3.10)	0.251	1. 18 (0.45, 3.07)	0.740
Education	Illiterate	1.00		1.00	
	Primary	2.71 (1.55, 4.75)	<0.001**	1.5 (0.72, 3.13)	0.275
	Secondary	5.42 (2.96, 9.92)	<0.001**	1.42 (0.62, 3.27)	0.404
	College and above	4.50 (2.02, 10.04)	<0.001**	1.23 (0.41, 3.66)	0.715
Family support	No	1.00		1.00	
	Yes	0.59(0.41, 0.84)	0.004**	0.53 (0.32, 0.87)	0.012*
Marital status	Married	1.00		1.00	
	Single	2.17 (1.48, 3.18)	<0.001**	1.06 (0.62, 1.84)	0.827
	Divorced/widowed	2.43 (1.24, 4.74)	0.009**	1.62 (0.67, 3.92)	0.286
Age for every 5 year increase		0.82 (0.75, 0.89)	<0.001**	0.97 (0.85, 1.11)	0.679
Duration of jail in year		0.96(0.89, 104)	0.320		
Residence	Rural	1.00		1.00	
	Urban	5.29 (3.51, 7.97)	<0.001**	2.15(1.20, 3.85)	0.010*
Jail history	No	1.00		1.00	
	Yes	2.18 (1.41, 3.36)	<0.001**	1.94 (1.09, 3.45)	0.024*
Health education	No	1.00			
	Yes	0.79 (0.51, 1.23)	0.304		
Sentence status	Not sentenced	1.00			
	Sentenced	087 (0.52, 1.45)	0.581		
HIV status	Negative	1.00			
	Positive	1.90 (0.57, 6.40)	0.299		
Alcohol use before jail	No	1.00		1.00	
	Yes	3.82(2.60, 5.62)	<0.001**	4.15(2.54, 6.79)	<0.001**
Multiple sexual partner	No	1.00		1.00	
	Yes	2.86(1.98, 4.12)	<0.001**	1.53 (0.95, 2.44)	0.079
Occupation	Civil servant	1.00			
	Farmer	0.20 (0.10, 0.38)	<0.001**	0.20 (0.08, 0.49)	<0.001**
	Merchant	1.62(0.73, 3.58)	0.237	0.92 (0.34, 2.52)	0.878
	Daily laborer	1.86(1.00, 3.43)	0.049*	1.08 (0.48, 2.42)	0.844
	Student	1.27(0.65, 2.46)	0.484	1.16 (0.49, 2.78)	0.737
	Drivers/soldiers/others	2.02(0.21, 0.56)	0.050*	2.13 (0.86, 5.28)	0.102

*Signifies for p-value <0.05.

** signifies for p-value<0.01.

On the other hand, we identified that urban residence (AOR = 2.15; 95%CI = 1.20, 3.84), previous history of incarceration (AOR = 1.91; 95%CI = 1.08, 3.40) and alcohol use before incarceration (AOR = 4.20; 95%CI = 2.57, 6.87) were significant determinants associated with higher odds of smoking. Moreover, prisoners who reported to have history of multiple sexual partners before their jail had a 47 per cent excess risk (AOR = 1.53; 95% CI = 1.95, 2.44) of smoking compared to prisoners who did not had multiple sexual partners, with border line significance (Table 3).

**Fig 2:**
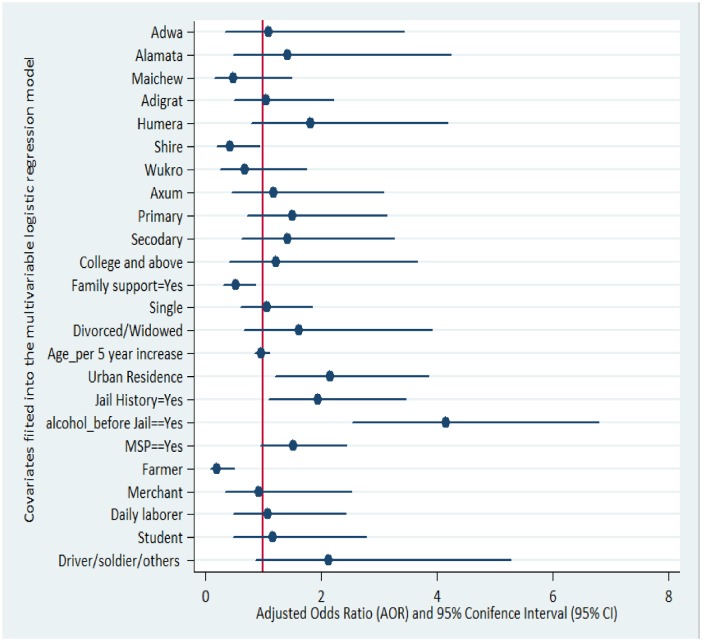
A forest plot of the adjusted effect of each fitted covariate on risk of smoking, estimated by odds ratio and 95% confidence interval, using multivariable binary logistic regression model.

Furthermore, increased odds of smoking were observed among prisoners who were drivers, soldiers, students, or daily labourers by occupation and similar risk was also observed among those who were single, divorced or widowed, but the difference was not statistically significant (Table 3). A forest plot of the multivariable model was produced to visualize the level and precision of association of each covariate with respect to smoking (Fig 2).

## Discussion

To summarize our key findings, the prevalence of smoking among prisoners with respiratory tract infection was 21 per cent. Results of this study suggest that a differentially increased burden of smoking was evident among coughing prisoners who had been incarcerated previously, who lived in urban areas and who drank alcohol. In contrast, prisoners who were farmers, jailed at Shire prison site and who received family support had differentially lower odds of smoking.

Seventy-one (8.8%) non-respondents were excluded from the main analysis for two methodological reasons. Firstly, they did not respond to the outcome of interest, smoking status, as a result of which analysis of these cases would not be methodologically possible. The second reason was that the non-response did not seem to be random when we explored according to socio-demographic, lifestyle and prison-related characteristics of the participants. In general, compared to the respondents, non-respondents were older people mostly living in rural areas with a farmer occupation and supported by their families. Nearly two-thirds of them did not relate their khat chewing status. The observed pattern has led us to assume that the non-response is in general systematic. In our multivariable model, we have found out that rural residence before jail, farmer occupation and older age are protective determinants. This compelling evidence, from our dataset, implies that exclusion of this non-respondent group could have overestimated the smoking prevalence. On the other hand, given the fact that the conservative culture of the rural Christian community of Tigray region against smoking and khat chewing, it is also possible that the non-respondents were smokers. Holding this assumption, one can think that our decision to exclude these non-respondents could have underestimated the smoking prevalence among prisoners in Tigray region. Nevertheless, our data are against this assumption and we rather conclude that exclusion of these non-respondents might have overestimated smoking prevalence.

The smoking prevalence of our study is lower than reports from previous studies conducted in prison setups [5,6,13–16]. The geographic variation could explain the observed difference in smoking burden among prisoners. Such variation could be due to socio-cultural, economic or policy differences. All the previous studies we used for comparison were conducted in developed countries where the smoking burden is expected to be higher compared to our setup. However, we boldly underline that our estimated burden of smoking is still a major health threat to the prisoners and their community and appropriate public health interventions should be established to address the problem we have uncovered.

Our results suggested that prisoners jailed at Shire prison had significantly lower odds of smoking compared to prisoners at the Mekelle site. Although we cannot fully explain the significantly lower risk of smoking in Shire, the high number of less risky population, as observed in our data compared to all sites, could partly explain the significantly lower risk, since no prison setting-based smoking policy or active intervention existed at any of the prison sites located in Tigray region. For instance, in Shire prison, 47.2 per cent of the participants were farmers and 91.2 per cent of the sample had no history of previous incarceration.

Our study has demonstrated that farmers had differentially significant lower odds of smoking compared to civil servants. This result was not different from our expectation, in that farmers most often live in rural areas where the conditions that predispose people to smoking are rarely available and where conditions that reduce smoking, such as more conservative religious practice, are common in the lives of the rural community. Our study has revealed that prisoners who received family support had a 47 per cent lower risk of smoking. This result was in line with other studies which demonstrated that family support was associated with lower risk of substance use and its predictors [16,20,21].

Another interesting result of our study was that prisoners with a history of previous incarceration had nearly two fold greater risk of smoking. The obtained result substantiates similar previous observations [13,14,18] and interventions that reduce criminal activities and incarceration could eventually break the cyclical risk of being a smoker. This highlights the need to implement interdisciplinary interventions targeting offending, which would be important to further prevent smoking in the vulnerable population. The significantly increased odds of smoking among those who reported an alcohol drinking habit before their incarceration was also demonstrated in other studies conducted with different populations and setups [22–24]. In one such study, it was mentioned that only seven per cent of smokers who drank alcohol were successful in their attempts to quit smoking, as compared to 49 per cent of the smokers who did not drink alcohol [24]. From our study, it is important to state that addressing associated factors, such as drinking alcohol, will have implications in addressing the problem of smoking in the jailed population.

Although our study provides useful insights on smoking from a vulnerable setting of a low income country, it has some limitations. Firstly, the study included prisoners who were suspected of TB and this could have affected its generalizability power by overestimating the smoking prevalence. Stated in other way, the exclusion of those prisoners who were not TB suspects might have limited the external validity of our study since risk of smoking could be higher among TB cases [25,26]. Secondly, our study neither did not take in to account smokeless tobacco use nor reliably quantified the amount of smoking. Our shortcoming of not considering smokeless tobacco use could have underestimated the true prevalence of smoking. The absence of a reliable quantification of the amount of smoking has limited important information that could give vital clue on the possible health effects of smoking among the participants and their inmates.

## Conclusion

Our study has guided us to conclude that smoking is a major health concern in prison setups located in Tigray region, northern Ethiopia. Our work has also given insight into the determinants of smoking among the prisoners, and interdisciplinary and comprehensive interventions should be developed to reduce the burden and consequences of smoking in the vulnerable population.

## Supporting Information

S1 Table This is the questionnaire of our study. (PDF)

## Abbreviations

AOR: adjusted odds ratio
CI: confidence interval
COR: crude odds ratio
