# Supplementary material for: Burden and Determinants of Smoking among Prisoners with Respiratory Tract Infection: A Cross-Sectional Study of Nine Major Prison Setups in Northern Ethiopia
Source: PLoS One. 2016 Dec 28;11(12):e0168941. doi: 10.1371/journal.pone.0168941 (PMC5193439; doi:10.1371/journal.pone.0168941)
Supplement: S1 Table — (PDF) [file pone.0168941.s001.pdf]

Date ------------------------

English version of smoking questionnaire

Questionnaire code number_________________ Prison name -----------------

Interviewer's Name______________________________Mobile No-----------------

Part I. Socio-demographic characteristics		
No	Questions	Coding category/response
1.	Age in years	------------years
2.	Sex	1. Female<br>2. Male
3.	Marital status	1. Single<br>2. Married<br>3. Divorced<br>4. Widowed<br>5. Separated
4.	Education	1. Not able to read and write<br>2. Able to read and write without formal school year<br>3. Primary (1-8)<br>4. Secondary (9-10 or 12)<br>5. College (10+ or 12+)<br>6. University degree
5.	Occupation before imprisonment	1. Civil servant(gov't)<br>2. Farmers<br>3. Merchant<br>4. Daily wages<br>5. Student<br>6. House wife<br>7. Unemployed<br>8. Others (specify__________________)
6.	Residence place (before detained)	1. Rural<br>2. Urban
7.	Have you ever smoked in the past 12 months?	1. Yes<br>2. No (If "No", skip to question 10)
8.	If your answer is "Yes" to question 7, how many packs/day?	_________ packs/day, _________cigarettes/day
9.	How long have you smoked?	______________(write the time unit in weeks or months or years)
10.	Do you chew 'khat'?	1. Yes<br>2. No (If "No", skip to question 12)
11.	If your answer is "Yes" to question 10, for how long have you chewed khat?	______________(write the time unit in weeks or months or years)

Date ------------------------

English version of smoking questionnaire

Questionnaire code number_________________ Prison name -----------------

Interviewer's Name_______________________________Mobile No-----------------

12.	Were you drinking alcohol including "Tela" and "Areki" (before your detention)?	1. Yes<br>2. No
13.	If your answer is "Yes" to question number 12, how frequent is it?	___________bottles per week for alcohol like beer<br>___________cups per week for areki or wiski
**Part II. Prison and health related aspects of prisoners**		
14.	Total number of prisoners in the prison site	____________prisoners
15.	Total number of rooms in the prison site	_____________rooms
16.	Total length of stay since detention	____________months/years (Write the unit of time)
17.	Type of prisoner	1. On court process<br>2. Sentenced
18.	How long have you been imprisoned in this prison site?	________(Write the time unit: months/ years)
19.	Have you ever been imprisoned previously?	1. Yes<br>2. No
20.	If your answer is "Yes" to question 19, how many times?	__________times
21.	Do you have support from your family in terms of visit and bringing food?	1. I don't have<br>2. Visit only<br>3. Food only<br>4. Both visit and food
22.	If you have family visit, how many times per week (month) do they bring you food?	____________times (per week or month or year)
23.	Do the prison health staff educate you about TB and other health related education?	1. Yes<br>2. No
24.	Do you have a cough?	1. Yes<br>2. No
25.	For how long have you been coughing?	------------weeks/months/years

Date ------------------------

English version of smoking questionnaire

Questionnaire code number_________________ Prison name -----------------

Interviewer's Name_______________________________Mobile No-----------------

26.	Currently, what kind of symptoms do you have on top of coughing?	1. difficulty of breathing<br>2. fever<br>3. weight loss<br>4. night sweating<br>5. loss of appetite<br>6. fatigue<br>7. bloody sputum<br>8. others (specify)__
27.	Time of occurrence of the cough	1. Before imprisonment<br>2. After imprisonment
28.	Did you visit and receive any treatment for your current complaint	1. Yes<br>2. No
29.	If your answer is "Yes" to question 28, where?	1. health institution outside of the prison<br>2. prison's clinic<br>3. both<br>4. Others (specify)------ -------------
30.	Do you have a diagnosed health problem like Diabetic mellitus, skin infections Hypertension…etc?	1. Yes<br>2. No
31.	Have you ever been hospitalized due to any health problem?	1. Yes<br>2. No
32.	If your answer is "Yes" to question 31, how long?	---------months
33.	If yes to question 31, what was the reason for your hospitalization?	………………………………………………………… ……………………………
34.	Have you ever had sexual intercourse out of your girl friend/boy friend or wife/ husband?	1. Yes<br>2. No
35.	If your answer is "Yes" to question number 61, have you been using condom consistently?	1. Yes<br>2. No
36.	Have you been tested for HIV?	1. Yes<br>2. No

Date ------------------------

English version of smoking questionnaire

Questionnaire code number__________________ Prison name ------------------

Interviewer's Name________________________________Mobile No------------------

37.	If your answer is "Yes" to question 36, what was the result?	1. Yes<br>2. No
38.	Current HIV serostatus (after HIV test)	1. Negative<br>2. Positive

Thank You So Much For Your Time and Contribution!!
